# Nonoperative Management Is Associated With an Increased Risk of Adverse Mental Health Conditions in Elderly Patients With Distal Radius Fractures: A Retrospective Cohort Analysis

**DOI:** 10.1177/15589447261489326

**Published:** 2026-10-08

**Authors:** Cameron J. Bowers, Jad Lawand, Patrick Tansey, Umar Ghilzai, John J. Faillace

**Affiliations:** 1 The University of Texas Medical Branch, Galveston, USA; 2 MacDill Air Force Base, Tampa, FL, USA; 3 Baylor College of Medicine, Houston, TX, USA

**Keywords:** distal radius, fracture/dislocation, diagnosis, forearm, anatomy, trauma, outcomes, research and health outcomes, psychosocial, surgery, specialty

## Abstract

**Background:**

Previous research comparing operative versus nonoperative management of distal radius fractures in elderly patients has reported similar functional outcomes, leaving treatment decisions controversial. However, limited studies have analyzed how these approaches impact the mental well-being of patients. The purpose of this study is to compare the incidence of mental health conditions during the 1-year post-injury period following operative and nonoperative treatment.

**Methods:**

A retrospective query of the TriNetX database identified patients 65 years and older with distal radius fractures who underwent open reduction with internal fixation (ORIF) within 1 month of diagnosis. These patients were 1:1 propensity score matched to a cohort treated nonoperatively with closed reduction for demographic factors and confounding comorbidities. Mental health outcomes within 1 year post-fracture were analyzed. Results were calculated as risk percentages, risk ratios (RRs), 95% confidence intervals (CIs), and *P* values.

**Results:**

Patients undergoing ORIF had a significantly lower risk of major depressive disorder (MDD) (1.7% vs 2.2%; RR, 0.78; 95% CI, 0.66-0.93; *P* = .01), selective serotonin reuptake inhibitor (SSRI) use (3.1% vs 4.0%; RR, 0.79; 95% CI, 0.69-0.90; *P* < .01), and opioid dependence (0.1% vs 0.2%; RR, 0.48; 95% CI, 0.27-0.88; *P* = .01) compared to the nonoperative cohort. There was no significant difference in the risk of depressive episodes, generalized anxiety disorder, or alcohol dependence.

**Conclusions:**

Operative fixation of closed distal radius fractures in elderly patients is associated with a lower incidence of new-onset perioperative mental health conditions, including MDD, SSRI use, and opioid dependence, compared with nonoperative treatment.

## Introduction

Distal radius fractures are among the most frequently managed fractures in health care. Court-Brown and Caesar^1^ found that distal radius fractures were responsible for 17.5% of all fractures, with a high prevalence among elderly patients, while Chung and Spilson^2^ reported that fractures of the distal radius were the second most common fractures occurring in elderly patients, behind hip fractures, with a projected incidence of 643 000 per year. The large volume of elderly patients experiencing these fractures is associated with a significant financial burden of treatment, with an estimated Medicare cost of $385 to $535 million annually.^3,4^ In addition, as life expectancy continues to increase and the number of elderly patients grows, the management of distal radius fractures in this population becomes increasingly important.

The initial management of stable distal radius fractures typically involves closed reduction with external casting or splinting followed by a period of immobilization; this treatment approach has demonstrated positive long-term functional outcomes in patients with fractures that have minimal to no displacement.^5,6^ However, unstable fractures that are at high risk for secondary displacement after initial reduction may require additional fixation. Displaced, unstable fractures are frequently fixed in young, active patients to restore anatomic alignment and improve functional outcomes.^7^ Multiple studies have shown beneficial functional outcomes following internal plate fixation in younger patients with regard to improved Disabilities of the Arm, Shoulder, and Hand (DASH) scores in the early postoperative period.^8-10^ However, studies in elderly patients show similar functional outcomes and DASH scores with either open reduction internal fixation (ORIF) or closed reduction of distal radius fractures.^11-14^ While there is not a clear improvement in functional outcomes in elderly patients undergoing ORIF, many elderly patients prefer operative management to achieve improved grip strength, earlier return to activity, and improved appearance.^15-17^

One gap that remains in the literature in the discussion between open surgical reduction and closed reduction in distal radius fractures in elderly patients is the mental health outcomes of patients undergoing these different treatment options. A review of patients with hip fractures found that 35% of elderly patients experience depression and anxiety.^18^ In addition, 20% of patients who experience orthopedic trauma meet the criteria for post-traumatic stress disorder.^19^ Bhandari et al^20^ reported that traumatized patients who develop depression or experience symptoms of psychological stress reported reduced quality of life metrics.

In orthopedic surgery, the success of treatment has often been measured by physical recovery with an emphasis on factors like pain relief, mobility, and structural repair. Yet growing evidence reveals that mental health profoundly shapes outcomes.^21-23^ Depression, anxiety, and psychosocial distress can influence pain perception, rehabilitation adherence, and surgical satisfaction. Understanding and addressing mental health in orthopedic populations are therefore not just complementary but essential to achieving true recovery and optimizing patient-centered care.

The purpose of this study is to compare the incidence of new mental health conditions in the early post-injury period following operative and nonoperative treatment in elderly patients with distal radius fractures. We hypothesized that operative fixation would be associated with a decreased incidence of new mental health conditions.

## Materials and Methods

This was a retrospective cohort study designed to evaluate mental health outcomes in elderly patients undergoing operative versus nonoperative management of distal radius fractures. This was achieved by utilizing TriNetX, a Health Insurance Portability and Accountability Act–compliant database that includes deidentified electronic medical records from 400 million patients from over 100 health care organizations. The US Collaborative Network of the database was queried on April 23, 2026, and included 67 participating health care organizations. Institutional review board approval was not required because this study uses completely deidentified patient information and did not involve the collection, use, or transmittal of individually identifiable data.

Using International Classification of Diseases, 10th Revision (ICD-10) and Current Procedural Terminology (CPT) codes, we identified 2 cohorts for evaluation (Supplemental Table S1). Cohort A was defined as patients who were 65 years of age or older who had a distal radius fracture and, within 1 month of diagnosis, had undergone ORIF of the fracture. Cohort B was defined as patients who were 65 years of age or older who had a distal radius fracture and, within 1 month of diagnosis, had undergone closed treatment of the fracture. Patients who had undergone ORIF were excluded from Cohort B. Inclusion criteria for both cohorts included patients who had management of their distal radius fractures done between April 22, 2015, and April 22, 2025, to ensure that patients had a minimum follow-up of 1 year prior to the time of analysis. Patients with open distal radius fractures were excluded from the analysis to ensure that only closed distal radius fractures were being compared between cohorts to reduce confounding.

The TriNetX platform was used to perform a 1:1 propensity score matching using logistic regression. The platform uses a greedy nearest-neighbor algorithm with a caliper of 0.1 pooled standard deviations. Propensity matching was used to balance demographics (age, sex, race, and ethnicity) and other potential confounders, including preoperative psychiatric diagnoses (psychosis, schizophrenia, and bipolar disorder), medical comorbidities that may affect management and outcomes (heart failure, chronic obstructive pulmonary disease, hypertension, tobacco use, obesity, and cerebrovascular diseases), and concurrent injuries (polytrauma, distal ulnar fractures, Galeazzi fractures, carpal fractures, and decompression fasciotomy for compartment syndrome) between the operative and nonoperative cohorts. The period for propensity matching included patient data from up to 1 year prior to the day of distal radius fracture.

All outcomes were analyzed after propensity score matching between 1 day and 1 year following distal radius fracture. Outcomes were evaluated using ICD-10 and National Library of Medicine Anatomical Therapeutic Chemical codes (Supplemental Table S1). The onset of acute mental health conditions evaluated were major depressive disorder (MDD), depressive episodes, generalized anxiety disorder, use of selective serotonin reuptake inhibitors (SSRIs), opioid dependence, and alcohol dependence. To reduce confounding, patients who also had these diagnoses prior to the time window were excluded from the analysis. Results were reported as risk percentages, risk ratios, 95% CIs, and *P* values. Results with a *P* value of less than .05 were considered significant. To further assess the clinical relevance of statistically significant outcomes, absolute risk reduction (ARR), relative risk reduction (RRR), and number needed to treat (NNT) values were calculated.

## Results

Before propensity score matching, significant differences were identified in demographic characteristics between cohorts. Patients undergoing operative treatment were more likely to be younger (71.9 ± 5.3 years vs 74.0 ± 6.1 years) and have concomitant distal ulnar fractures (43.4% vs 34.8%) and carpal fractures (30.1% vs 23.1%). In addition, there were significantly more patients who underwent operative treatment with hypertension (50.5% vs 44.3%) and obesity (12.1% vs 6.8%) and fewer with heart failure (5.1% vs 7.4). However, after 1:1 matching, there were no significant differences in demographics or the potential confounding factors assessed between the operative and nonoperative cohorts (Table 1).

**Table 1.** Cohort Demographics Before and After 1:1 Propensity Matching of Operative Treatment Versus Nonoperative Treatment of Distal Radius Fractures.

Demographics	Unmatched cohort			Matched cohort		
	Operative treatment N = 18 300	Nonoperative treatment N = 24 377	Standard mean difference	Operative treatment N = 16 394	Nonoperative treatment N = 16 394	Standard mean difference
	n (%)	n (%)		n (%)	n (%)	
Age^a^	71.9 (5.3)	74.0 (6.1)	0.38	72.3 (5.3)	72.2 (5.4)	0.01
Male	2573 (14.1)	3788 (15.5)	0.04	2381 (14.5)	2346 (14.3)	0.01
Female	15 726 (85.9)	20 582 (84.4)	0.04	14 012 (85.5)	14 047 (85.7)	0.01
White	15 986 (87.4)	20 847 (85.5)	0.05	14 265 (87.0)	14 253 (86.9)	<0.01
African American	632 (3.5)	1124 (4.6)	0.06	585 (3.6)	582 (3.6)	<0.01
Asian	671 (3.7)	809 (3.3)	0.02	596 (3.6)	598 (3.6)	<0.01
Unknown race	579 (3.2)	928 (3.8)	0.04	545 (3.3)	558 (3.4)	<0.01
Other race	317 (1.7)	508 (2.1)	0.03	302 (1.8)	315 (1.9)	0.01
Hispanic or Latino	1003 (5.5)	978 (4.0)	0.07	802 (4.9)	779 (4.8)	0.01
Not Hispanic or Latino	13 705 (74.9)	18 526 (76.0)	0.03	12 359 (75.4)	12 353 (75.4)	<0.01
Unknown ethnicity	3592 (19.6)	4873 (20.0)	0.01	3233 (19.7)	3262 (19.9)	<0.01
Polytrauma	242 (1.3)	276 (1.1)	0.02	207 (1.3)	196 (1.2)	0.01
Distal ulnar fracture	7937 (43.4)	8493 (34.8)	0.18	6603 (40.3)	6515 (39.7)	0.01
Galeazzi fracture	279 (1.5)	240 (1.0)	0.05	201 (1.2)	202 (1.2)	<0.01
Carpal fracture	5509 (30.1)	5619 (23.1)	0.16	4461 (27.2)	4380 (26.7)	0.01
Decompression fasciotomy	17 (0.1)	0 (0.0)	0.04	10 (0.1)	0 (0.0)	0.04
Schizophrenia	29 (0.2)	73 (0.3)	0.03	28 (0.2)	33 (0.2)	0.01
Psychosis	14 (0.1)	35 (0.1)	0.02	14 (0.1)	14 (0.1)	<0.01
Bipolar disorder	150 (0.8)	203 (0.8)	<0.01	139 (0.8)	128 (0.8)	0.01
Heart failure	929 (5.1)	1813 (7.4)	0.10	886 (5.4)	818 (5.0)	0.02
Chronic obstructive pulmonary disease	1570 (8.6)	2135 (8.8)	0.01	1409 (8.6)	1369 (8.4)	0.01
Hypertension	9238 (50.5)	10 806 (44.3)	0.12	7871 (48.0)	7773 (47.4)	0.01
Tobacco use	262 (1.4)	339 (1.4)	<0.01	238 (1.5)	214 (1.3)	0.01
Overweight and obesity	2209 (12.1)	1667 (6.8)	0.18	1492 (9.1)	1447 (8.8)	0.01
Cerebrovascular disease	1116 (6.1)	1840 (7.5)	0.06	1034 (6.3)	955 (5.8)	0.02

Abbreviations: n, number of patients; %, percentage of patients.

a Data are presented as mean (standard deviation).

Prior to propensity matching, there were 18 300 patients in the operative treatment group and 24 377 patients in the nonoperative treatment group. In the matched cohort, 16 394 patients were included in each group for analysis. Patients who underwent ORIF experienced a significantly lower risk of an acute diagnosis of MDD (1.7% vs 2.2%; RR, 0.78; 95% confidence interval [CI], 0.66-0.93; *P* = .01), SSRI use (3.1% vs 4.0%; RR, 0.79; 95% CI, 0.69-0.90; *P* < .01), and opioid dependence (0.1% vs 0.2%; RR, 0.48; 95% CI, 0.27-0.88; *P* = .01) compared with the nonoperative management cohort within 1 year post-fracture (Table 2). There was no significant difference in the risk of a new-onset diagnosis of depressive episodes (3.0% vs 3.5%; RR, 0.87; 95% CI, 0.76-1.00; *P* = .05), generalized anxiety disorder (0.9% vs 1.1%; RR, 0.82; 95% CI, 0.66-1.04; *P* = .10), or alcohol dependence (0.2% vs 0.3%; RR, 0.84; 95% CI, 0.54-1.30; *P* = .42) between groups (Figures 1 and 2).

**Table 2.** One-Year Post-Fracture Outcome Evaluation After Propensity Matching Between Operative and Nonoperative Treatment of Distal Radius Fractures.

Outcome	Operative treatment N = 16 394	Nonoperative treatment N = 16 394	RR (operative: nonoperative treatment)	95% CI	*P* value
	n (%)	n (%)			
Major depressive disorder	235 (1.7)	289 (2.2)	0.78	0.66-0.93	.01
Depressive episode	384 (3.0)	425 (3.5)	0.87	0.76-1.00	.05
Generalized anxiety disorder	133 (0.9)	159 (1.1)	0.82	0.66-1.04	.10
SSRI use	405 (3.1)	500 (4.0)	0.79	0.69-0.90	<.01
Opioid dependence	16 (0.1)	33 (0.2)	0.48	0.27-0.88	.01
Alcohol dependence	37 (0.2)	44 (0.3)	0.84	0.54-1.30	.42

Abbreviations: CI, confidence interval; n, number of patients; RR, risk ratio; SSRI, selective serotonin reuptake inhibitor; %, percentage of patients.

**Figure 1.**
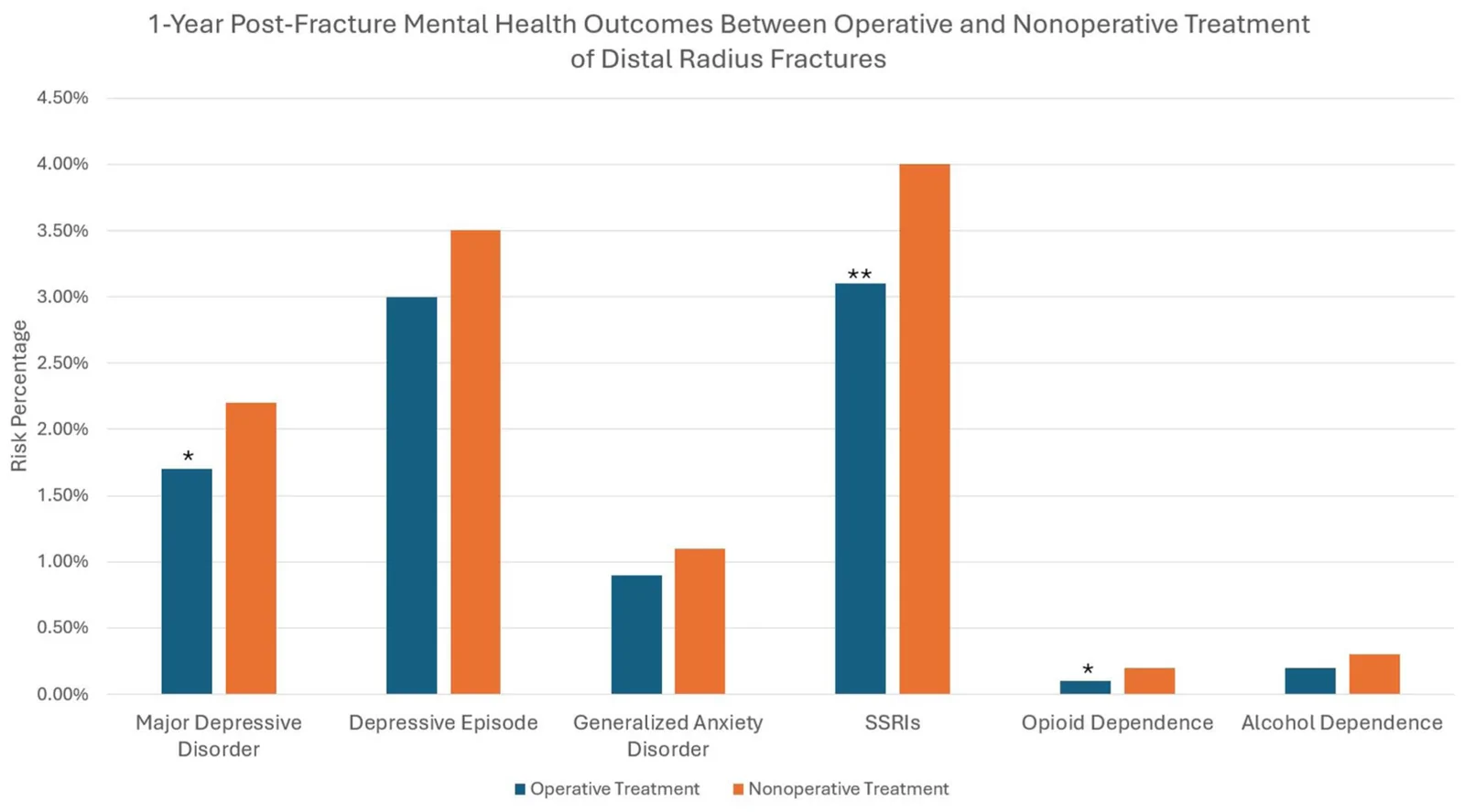
Graph showing cohort outcome assessment after propensity score matching between operative (blue) versus nonoperative (orange) management of distal radius fractures. SSRI indicates selective serotonin reuptake inhibitor. *P < .05, **P < .01.

**Figure 2.**
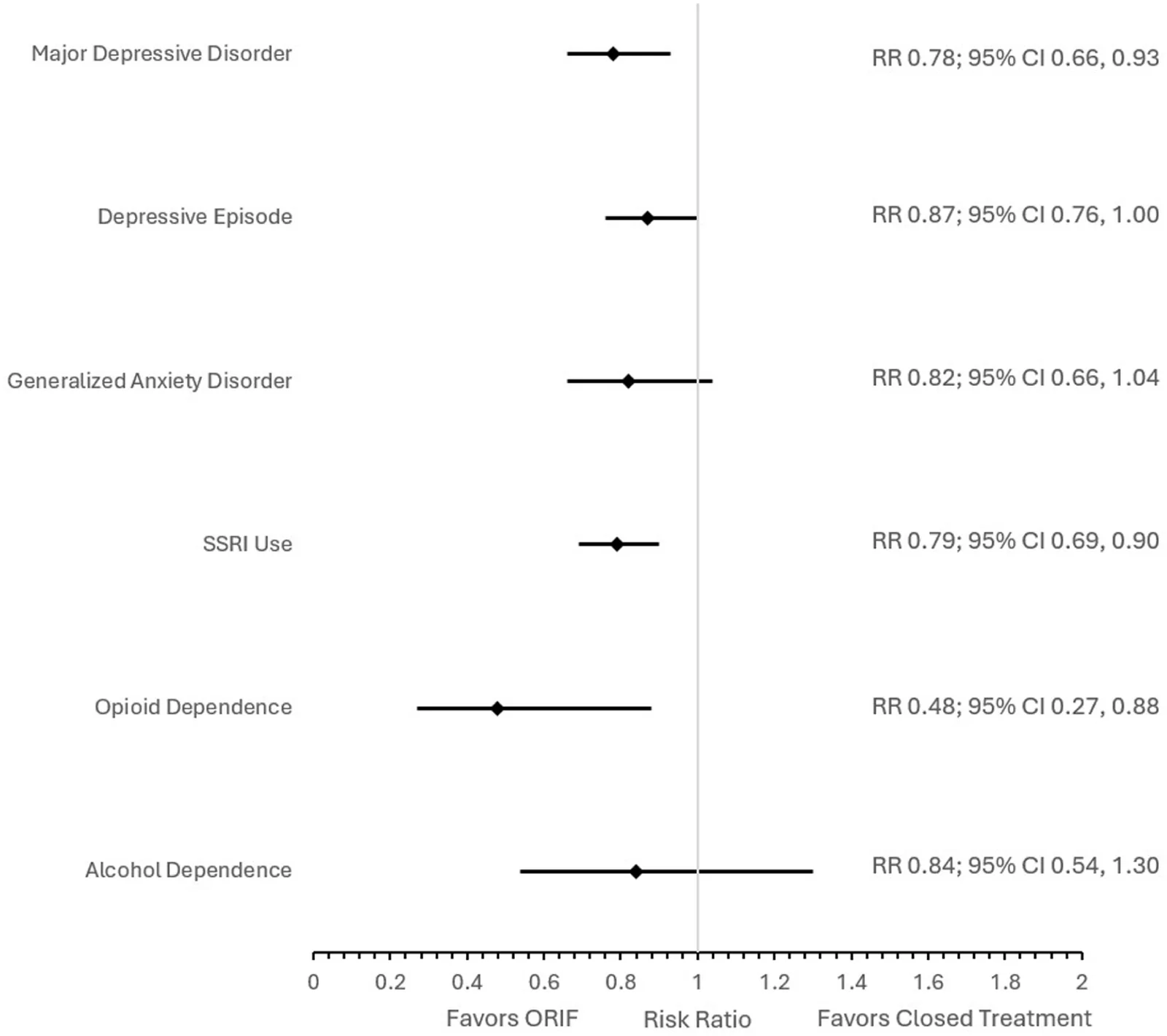
Forest plot showing risk ratio (RR) and 95% confidence intervals (CIs) for operative versus nonoperative treatment. ORIF indicates open reduction internal fixation; and SSRI, selective serotonin reuptake inhibitor.

For results that were found to be statistically significant, we further evaluated their clinical relevance by calculating the ARR, RRR, and NNT based on their risk percentage and relative risk values. The corresponding values for MDD were an ARR of 0.5%, an RRR of 22%, and an NNT of 200 patients. With SSRI use, there was an ARR of 0.9%, an RRR of 21%, and an NNT of 112 patients. Finally, for opioid dependence, there was an ARR of 0.1%, an RRR of 52%, and an NNT of 1000 patients (Table 3).

**Table 3.** Clinical Relevance of Statistically Significant Mental Health Outcomes Reported as Absolute Risk Reduction (ARR), Relative Risk Reduction (RRR), and Number Needed to Treat (NNT) Values.

Outcome	ARR (nonoperative control event rate − operative treatment event rate)	RRR (1 − relative risk)	NNT (1/ARR)
Major depressive disorder	0.5%	22%	200
SSRI use	0.9%	21%	112
Opioid dependence	0.1%	52%	1000

Abbreviations: ARR, absolute risk reduction; NNT, number needed to treat; RRR, relative risk reduction; SSRI, selective serotonin reuptake inhibitor.

## Discussion

The main finding of this study was that there was an association between elderly patients undergoing ORIF of distal radius fractures and a lower incidence of an acute diagnosis of mental health conditions, including MDD, SSRI use, and opioid dependence, within a 1-year period compared to patients who had a closed reduction. Regarding the clinical significance of these findings, although the ARRs observed in this study were modest, the findings may be clinically relevant when interpreted in a population-level context. Operative fixation was associated with ARRs of 0.5% to 0.9% for MDD and SSRI initiation, with corresponding NNTs of 200 and 112 patients. In addition, there were relative risk reductions of 22% and 21% for the acute incidence of MDD and SSRI use. This reduction is important as these outcomes have been associated with substantial morbidity, impaired functional recovery, and increased health care utilization in elderly patients. Furthermore, even at lower incidence rates, the reduced risk of opioid use is notable given the severity and long-term consequences of opioid use disorders. While these differences may not represent large treatment effects individually, they suggest potentially meaningful benefits at a population level in a high-volume injury such as distal radius fractures. Conversely, there was no significantly reduced risk in the acute incidence of depressive episodes, anxiety, or alcohol dependence. Overall, these results should be interpreted as hypothesis generating and support incorporating mental health considerations into shared decision-making, rather than as prescriptive indications for operative management.

When comparing the acute incidence of these mental health conditions in our study to the prevalence of these conditions in the population at large, the values from our analysis tend to be lower. Hasin et al^24^ conducted a national survey of 36 309 adults in the United States and reported a 12-month prevalence of MDD of 10.4%. Conversely, our study found a 12-month incidence of 1.7% and 2.2% among elderly patients with acute-onset MDD following operative versus nonoperative treatment of distal radius fractures. Furthermore, previous studies have reported a 12-month prevalence of SSRI use of approximately 12% to 15% in the general population in the United States.^25^ However, our study showed a 12-month incidence of 3.2% and 3.9% among elderly patients prescribed SSRIs following operative versus conservative management of distal radius fractures. In addition, the 2015 National Survey on Drug Use and Health, completed by 51 200 adults, reported opioid use disorder in 0.8% of participants in the prior year.^26^ However, we found an incidence of 0.1% and 0.2% of opioid dependence in elderly patients over a 12-month period following operative and nonoperative treatment of distal radius fractures. This trend of having a lower incidence of these mental health conditions than the 12-month prevalence in the general population may be explained by several factors. For example, our study excluded patients who already had a prior diagnosis of MDD, SSRI use, or opioid dependence. In addition, the prevalence of MDD has been reported to be significantly lower in elderly patients compared to younger adults.^24^ Also, previous literature has reported a sustained decrease in SSRI prescriptions with age, particularly in elderly men.^27^

These findings provide an additional perspective on the ongoing debate regarding management of distal radius fractures in this population in a way that has not previously been analyzed. Much of the prior literature has focused on functional outcomes of patients with measures such as DASH and Patient-Rated Wrist Evaluation (PRWE) scores. One prospective randomized study of 73 elderly patients with displaced and unstable distal radius fractures found that patients who underwent ORIF with a volar locking plate (VLP) had lower DASH and PRWE scores in the early postoperative period compared to patients who were treated with closed reduction and casting; however, there were no significant differences in DASH or PRWE scores at 6 or 12 months postoperatively.^12^ Their study also found significantly improved grip strength in patients who underwent operative fixation compared to nonoperative management. One hypothesis that may contribute to our findings of an associated reduced incidence of adverse mental health outcomes with operative management is that improved DASH and PRWE scores in the early postoperative period, as well as superior grip strength, may contribute to higher patient satisfaction and functional independence, reducing their likelihood of developing mental health conditions.

Other larger-scale studies, including systematic reviews and meta-analyses, have concluded that operative treatment with VLPs is not associated with improved DASH scores or other functional outcomes compared to nonsurgical treatment in patients older than 60 years.^11,14,28^ As there is continued debate over the treatment of distal radius fractures in elderly patients, the decision is often weighed based on the surgeon’s preference and training background. It has been previously reported that hand fellowship–trained surgeons are significantly more likely to treat with internal fixation than surgeons who did not complete hand fellowship training.^29^

Although the consensus of previous studies is that there are no significant differences in DASH scores in elderly patients undergoing either ORIF or closed reduction of distal radius fractures, some have noted other advantageous outcomes in favor of surgical management. For example, a systematic review and meta-analysis done by Li et al^11^ found that elderly patients treated with VLPs had significantly better grip strength and radiographic assessment compared to the nonoperative group. It is well accepted that ORIF of distal radius fractures allows patients to achieve earlier range of motion and weight-bearing compared to nonoperative treatment. The combination of grip strength, range of motion, and ability to bear weight all contribute to function. The delay in range of motion and weight-bearing status in nonoperative treatment could contribute to loss of muscle mass. Prior studies have indicated that loss of muscle mass is associated with loss of physical function as scored by the Patient-Reported Outcomes Measurement Information System, which may reduce a patient’s ability to perform activities of daily living.^30^ Functional independence is known to be of particular importance in elderly patients, with prior studies showing that this is associated with improved mental health outcomes.^31,32^ This may contribute to the preference of elderly patients to undergo operative fixation of distal radius fractures.^33^ Some of these findings from prior studies influenced the development of our hypothesis that elderly patients receiving operative fixation would be associated with a decreased incidence of acute mental health conditions. It was theorized that some of these beneficial outcomes, such as improved grip strength, would reduce the risk of adverse mental health conditions with the assumption that these factors may be associated with improved quality of life and patient satisfaction. In addition, patients who favor operative management may be more active with higher demand at baseline; as such, these patients may be less prone to develop depression or other mental health issues.

Comparison of functional outcomes is certainly important in the assessment of whether to pursue operative or nonoperative treatment; however, it may not capture the entirety of postoperative outcomes. Prior research comparing surgical versus nonsurgical intervention for other fractures in elderly patients has considered variables beyond functional outcomes. For example, Loggers et al evaluated quality of life in patients older than 70 years undergoing operative or nonoperative treatment of proximal femoral fractures using EuroQol 5-Dimension 5-Level (EQ-5D-5L) scores as their primary outcome. They found that the patients undergoing operative or nonoperative treatment had similar EQ-5D-5L scores.^34^ The lack of similar studies that evaluate quality-of-life outcomes in elderly patients undergoing ORIF versus closed treatment of distal radius fractures stimulated the aim of this study to assess mental health outcomes in these patients.

### Limitations and Future Directions

While this study provides valuable insights into mental health outcomes in elderly patients undergoing operative versus nonoperative treatment of distal radius fractures, it has some limitations. The reliance on the TriNetX database may not include all elderly patients with distal radius fractures or their subsequent treatment and mental health outcomes, as some may seek care in other health care settings that are not participating organizations in the database. Furthermore, the results are dependent on accurate entry by health care providers. In addition, the study lacks further detail on the nature of the distal radius fractures with regard to their fracture patterns, stability, and severity. These factors would also be important with regard to clinical decision-making for operative versus nonoperative treatment. Other factors that may influence decision-making for operative or conservative management and postoperative mental health conditions are medical comorbidities. While we attempted to reduce this by matching for medical comorbidities, this is limited by the granularity of using ICD-10 codes for conditions without capturing their severity at the time of treatment.

Furthermore, there are various other factors that may influence mental health outcomes that are not accounted for by the database. For example, socioeconomic status, living situation, and social support systems are all variables that could affect mental health outcomes in these patients that cannot be controlled by using the database. Future prospective studies in elderly patients undergoing operative versus nonoperative treatment of distal radius fractures and using other methods to gain a broader understanding of mental health and quality of life in these patients are indicated.

## Conclusions

In our study, operative fixation of closed distal radius fractures in elderly patients is associated with a lower incidence of new-onset perioperative mental health conditions, including MDD, depressive episodes, SSRI use, and opioid dependence compared with nonoperative treatment. Taking the well-being of patients and their mental health status following treatment into consideration may be valuable in the decision between surgical versus nonsurgical intervention for distal radius fractures in elderly patients.

## Supplemental Material

sj-docx-1-han-10.1177_15589447261489326 – Supplemental material for Nonoperative Management Is Associated With an Increased Risk of Adverse Mental Health Conditions in Elderly Patients With Distal Radius Fractures: A Retrospective Cohort Analysis Supplemental material, sj-docx-1-han-10.1177_15589447261489326 for Nonoperative Management Is Associated With an Increased Risk of Adverse Mental Health Conditions in Elderly Patients With Distal Radius Fractures: A Retrospective Cohort Analysis by Cameron J. Bowers, Jad Lawand, Patrick Tansey, Umar Ghilzai and John J. Faillace in HAND

## Data Availability

All data generated or analyzed during this study are included in this published article (and its supplementary information files).
