# Supplementary material for: Nonoperative Management Is Associated With an Increased Risk of Adverse Mental Health Conditions in Elderly Patients With Distal Radius Fractures: A Retrospective Cohort Analysis
Source: Hand (N Y). 2026 Oct 8:15589447261489326. Online ahead of print. doi: 10.1177/15589447261489326 (PMC13652978; doi:10.1177/15589447261489326)
Supplement: Supplementary file 1 [file sj-docx-1-han-10.1177_15589447261489326.docx]

Supplemental Table S1. Coding systems and codes used for all variables included in cohort definitions, propensity score matching, and data analysis.

| **Descriptor** | **Codes Utilized** |
| --- | --- |
| **Cohort Definitions** |  |
| Distal radius fracture | ICD-10: S52.5 |
| Open reduction with internal fixation of distal radius fractures | CPT: 25607, 25608, 25609 |
| Closed treatment of distal radius fractures | CPT: 25600, 25605 |
| Open distal radius fractures | ICD-10: S52.501B, S52.501C, S52.502B, S52.502C, S52.509B, S52.509C |
|  |  |
| **Propensity Matching** |  |
| Polytrauma | ICD-10: T07.XXXA |
| Distal ulnar fracture | ICD-10: S52.6 |
| Galeazzi’s fracture | ICD-10: S52.37 |
| Carpal fracture | ICD-10: S62.1 |
| Decompression fasciotomy | CPT: 25020 |
| Schizophrenia | ICD-10: F20 |
| Psychosis | ICD-10: F29 |
| Bipolar disorder | ICD-10: F31 |
| Heart failure | ICD-10: I50 |
| Chronic obstructive pulmonary disease | ICD-10: J44 |
| Essential hypertension | ICD-10: I10 |
| Tobacco use | ICD-10: Z72.0 |
| Overweight and obesity | ICD-10: E66 |
| Cerebrovascular disease | ICD-10: I60-I69 |
|  |  |
| **Outcomes** |  |
| Major depressive disorder | ICD-10: F32.9 |
| Depressive episode | ICD-10: F32 |
| Generalized anxiety disorder | ICD-10: F41.1 |
| SSRI use | NLM:ATC:N06AB |
| Opioid Dependence | ICD-10: F11.2 |
| Alcohol Dependence | ICD-10 F10.2 |

Key: ICD-10, International Classification of Diseases, 10^th^ edition; CPT, current procedural terminology; NLM:ATC, National Library of Medicine Anatomical Therapeutic Chemical; SSRI, selective serotonin reuptake inhibitor.
